# The onset of electrospray: the universal scaling laws of the first ejection

**DOI:** 10.1038/srep32357

**Published:** 2016-09-01

**Authors:** A. M. Gañán-Calvo, J. M. López-Herrera, N. Rebollo-Muñoz, J. M. Montanero

**Affiliations:** 1 Depto. de Ingeniería Aeroespacial y Mecánica de Fluidos, Universidad de Sevilla, E-41092 Sevilla, Spain; 2 Depto. de Ingeniería Mecánica, Energética y de los Materiales and Instituto de Computación Científica Avanzada (ICCAEx), Universidad de Extremadura, E-06006 Badajoz, Spain

## Abstract

The disintegration of liquid drops with low electrical conductivity and subject to an electric field is investigated both theoretically and experimentally. This disintegration takes place through the development of a conical cusp that eventually ejects an ultrathin liquid ligament. A first tiny drop is emitted from the end of this ligament. Due to its exceptionally small size and large electric charge per unit volume, that drop has been the object of relevant recent studies. In this paper, universal scaling laws for the diameter and electric charge of the first issued droplet are proposed and validated both numerically and experimentally. Our analysis shows how charge relaxation is the mechanism that differentiates the onset of electrospray, including the first droplet ejection, from the classical steady cone-jet mode. In this way, our study identifies when and where charge relaxation and electrokinetic phenomena come into play in electrospray, a subject of live controversy in the field.

Understanding the effects produced by electric fields on capillary systems is of great importance in a large number of applications including biochemical analysis, inkjet printing, spray coating, electrowetting, fabrication of microspheres of biological materials, direct handling of living cells, etc. For instance, the current research in analytical chemistry (mass spectrometry) is exploring new ultra-sensitive technologies for sample disintegration and charging, whose fundamentals reside in electrohydrodynamic atomization processes^1^.

Under certain conditions, a low-conductivity^2^,^3^ drop subject to a strong electric field ejects a thin liquid filament, which issues a stream of tiny charged drops. The distribution of the individual masses and charges in the line of droplets is essentially determined by the liquid properties. This distribution depicts a unique signature of the ejection process, reflecting the unsteady electrohydrodynamic history leading to them. The first ejected droplet is particularly important because of its exceptionally small size and large electric charge per unit volume. The full understanding of this phenomenon will enable complete command of the liquid ejecta involved in the different modes of electrospraying, including pulsating regimes, and will open new avenues for the conception of innovative technologies.

Fontelos *et al.*^4^ studied theoretically the unsteady elementary disintegration of charged and neutral conducting drops under externally applied electric fields. Their primary interest was the evolution of the drops until they develop conical tips. Depending on the magnitude of the applied electric field, the drop produces a single cusp, two opposite tips, or multiple peaks^5^. Those tips eventually beget ultra-thin liquid jets. The tip streaming arising in low-conductivity drops after a step change in the electric field magnitude has been examined both numerically^6^,^7^,^8^ and experimentally^8^,^9^,^10^,^11^. The understanding of the origin of such a singularity and the determination of its parameter constraints is important. However, these aspects of the problem lag behind most applications, where the major interest is precisely the ejecta.

Collins *et al.*^6^,^7^ focused on the deformation of a capillary free surface and the subsequent droplet ejection when a sufficiently strong electric field is suddenly applied. Their pioneering investigations produced a series of very accurate numerical simulations using the Taylor-Melcher leaky-dielectric model^3^, assuming charge relaxation at the free surface (no net charge in the bulk). In ref. 7, they studied the disintegration of a low-conductivity suspended drop. The electric field eventually caused the ejection of mass and charge from two symmetrical cusps oriented in the field direction. They proposed universal scaling laws for both the size and charge of the first emitted droplet.

The assumptions involved in the leaky-dielectric model as related to general unsteady electrohydrodynamic phenomena were discussed in depth by Schnitzer and Yariv^12^. These assumptions have two consequences. On one side, the net volumetric charge density in the hydrodynamic bulk is assumed sufficiently small for the dynamical effects produced by electrostatic forces to be negligible in that region. For this reason, attention is paid exclusively to the charge accumulated on the interface and the resulting Maxwell stresses. On the other side, the surface charge density is determined by integrating a surface charge conservation equation, where the transfer of ions from the bulk is calculated in the same manner as in the steady regime. Specifically, the local variation of surface charge density is proportional to *KE*_*n*_, where *K* is the electric conductivity (measured on steady conditions) and *E*_*n*_ the normal component of the electric field evaluated on the liquid side of the free surface. This calculation implies that the bulk becomes a non-depletable source of charge, which provides, wherever necessary, a net flux *KE*_*n*_. While the first part of this twofold assumption may generally constitute a good approximation for problems with low-conductivity liquids, we will show how the second fails in highly-unsteady and ultra-fast liquid disintegration. In this case, the charge migration towards the interface is limited by the ion mobility, which becomes comparable to the interface speed.

The surface charge conservation equation described above does not reflect the physics of the highly-unsteady liquid disintegration. The analysis of such a phenomenon requires a different approach, where electric charge is traced during its evolution throughout the liquid. Very recently^13^, Pillai *et al.* have recognized this fact in studying the transient electrohydrodynamic response of liquid microdrops with small electric conductivities subject to externally applied electric fields (note: present work was conducted one year prior to the publication of ref. 13). In that study, the droplet radius was comparable to the Debye length, and therefore the droplet ejection was influenced by thermal diffusion of electrical charge. Their results indicate that the unsteady charge transport across the bulk significantly affects the ejection of the progeny droplets for large enough applied electric fields. Due to this effect, their scaling laws for the diameter and charge of the issued droplet differ substantially from those derived by Collins *et al.*^7^ from the leaky-dielectric model.

In this paper, we provide a revision of the electrohydrodynamic process responsible for the electrospray onset. We find that the first-droplet ejection is essentially driven by charge relaxation, which invalidates the leaky-dielectric assumption described above^6^,^7^. This effect makes the process fundamentally different from steady electrospray, which relies on complete charge relaxation. As will be seen, our analysis is valid for negligible thermal diffusion, zero net production of electrical charge, and negligible dynamical effects of the outer medium. However, it does not resort to the small-size-drop requisite (as occurs in ref. 13) to show the relevance of charge relaxation in electrical tip streaming. Even if the Debye length is much smaller than the system characteristic size, and therefore diffusion effects can be neglected, the ejecta are affected by the charge transport driven by the electrical field. Our results lead to universal scaling laws for both the size and charge of the first-issued droplet, fostering the understanding of the whole electrospray phenomenon.

## Results and Discussion

The formation of the liquid thread begetting the first-ejected droplet is a local process which takes place in a region much smaller than the parent drop. Therefore, one can assume that all the geometrical variables characterizing the boundary conditions (e.g., the radius of the disk/capillary on which the parent drop hangs) do not significantly affect this process^7^. In addition, both numerical simulations and experiments have shown that the applied electric field must lie within a relatively narrow range for the ejection to occur, and therefore that quantity has little influence on the jet formation once that condition verifies^14^,^15^. As will be justified in *Methods*, both thermal diffusion and net production of electrical charge can be neglected under quite general conditions. Finally, we shall assume that the environment is a low density and viscosity dielectric fluid, and thus its dynamical effects can be neglected too.

If one takes into account the above approximations, the parameters which essentially characterize the problem are the liquid properties (density *ρ*, viscosity *μ*, surface tension *σ*, electrical conductivity *K*, and electrical permittivity *ε*_*i*_) and the electrical permittivity *ε*_*o*_ of the enviroment (vacuum or gas). From these parameters, one defines the characteristic length 
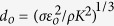
, time *t*_*o*_ = *ε*_*o*_/*K*, velocity *v*_*o*_ = (*σK*/*ρε*_*o*_)^1/3^, electric field magnitude^14^ *E*_*o*_ = (*σ*/*d*_*o*_*ε*_*o*_)^1/2^, and the electric charge 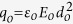. The problem is finally governed by the two dimensionless numbers^16^,^17^ *δ*_*μ*_ = *ρd*_*o*_*v*_*o*_/*μ* and *β* = *ε*_*i*_/*ε*_*o*_. The dimensionless diameter *d*/*d*_*o*_ and electric charge *q*/*q*_*o*_ of the first-ejected droplet depend on those two numbers exclusively.

The equations for the balance of mass, momentum, energy, and electric charge taking place in the cone-jet region of the *steady* electrospray mode lead to a set of scaling laws which successfully describe this regime over a wide range of experimental conditions^18^. However, and as will be shown, the present problem presents a fundamental feature associated to its inherent *unsteadiness*, which invalidates those balance equations: the dominant role played by charge relaxation.

**Figure 1:**
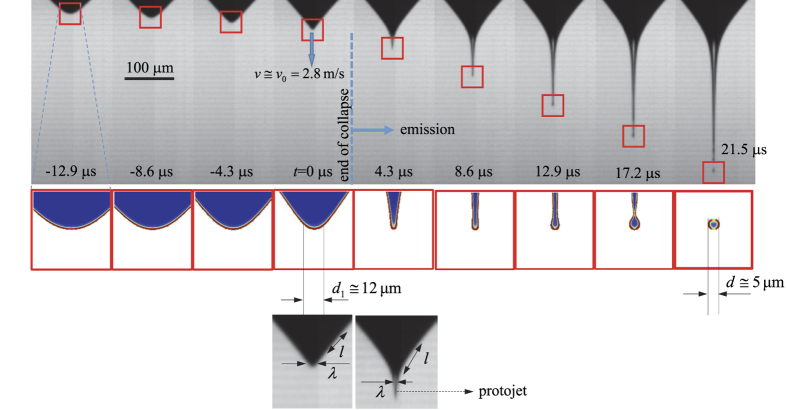
Global view of the first-droplet ejection process. The upper images correspond to an experiment with 1-Octanol. The central graphs show the evolution of the front from the corresponding numerical simulations (see *Methods*). The colors indicate the dimensionless volume charge density 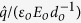 from 0 (blue) to 1 (red). The two lower images show the magnification of the meniscus tip for *t* = 0 and *t* = 4.3 *μ*s. The process can be divided into two stages: the droplet tip collapse for *t* < 0, and the jet emission for *t* > 0.

In this section, we show that the first-droplet ejection process relies upon three key features: (a) the self-similar collapse of the drop’s tip with a velocity independent from both viscosity and permittivity, (b) the lack of full charge relaxation from the bulk to the free surface due to the large velocities reached in the tip collapse, which fixes the normal electric field on the surface, and (c) the balance between inertia, axial viscous stresses, surface tension, and electrostatic suction during the jet’s evolution. Figure 1 shows a global view of the first-droplet ejection process and the relevant dimensional parameters introduced below. This process can be split into two stages: the droplet tip collapse for *t* < 0, and the jet emission for *t* > 0.

### Self-similar collapse of the drop’s tip

The liquid ejection is initiated by the deformation of the parent drop tip. Consider a certain instant of the collapse and early emission, and the portion of the drop’s tip delimited by a reference cross-section with a radial characteristic size *λ*. The electric current *I* conducted towards that portion can be calculated as *I* ~ *Kλ*^2^*E*_*z*_, where *E*_*z*_ ~ (*σ*/*ε*_*o*_*l*)^1/2^ is the axial electric field due to the quasi-electrostatic Taylor cone shaped along the process^18^, and *l* is an axial characteristic length of the cone. The instantaneous electric power *W* provided by the applied voltage is *W* ~ *I*Δ*V*, where Δ*V* ~ *E*_*z*_*l* is the electric potential decay along the distance *l*. This power is converted mainly into kinetic energy, whose increase per unit time is on the order of the flux *ρv*^3^*λ*^2^ across the reference cross-section, where *v* is the characteristic axial velocity in the collapse of the drop’s tip. Taking into account the above expressions, energy conservation demands 
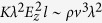
, which yields


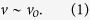


As can be seen, the characteristic axial velocity *v* becomes independent of both viscosity and permittivity, and coincides with that obtained for steady electrospray^16^,^18^ because the conversion of electric potential into kinetic energy follow the same route in both cases. In addition, the auxiliary characteristic lengths *λ* and *l* neatly cancel out from the analysis. These are *instantaneous geometrical features* that change from one instant to another (Fig. 1) without affecting the result obtained, which implies that the ejection process exhibits self-similarity.

**Figure 2:**
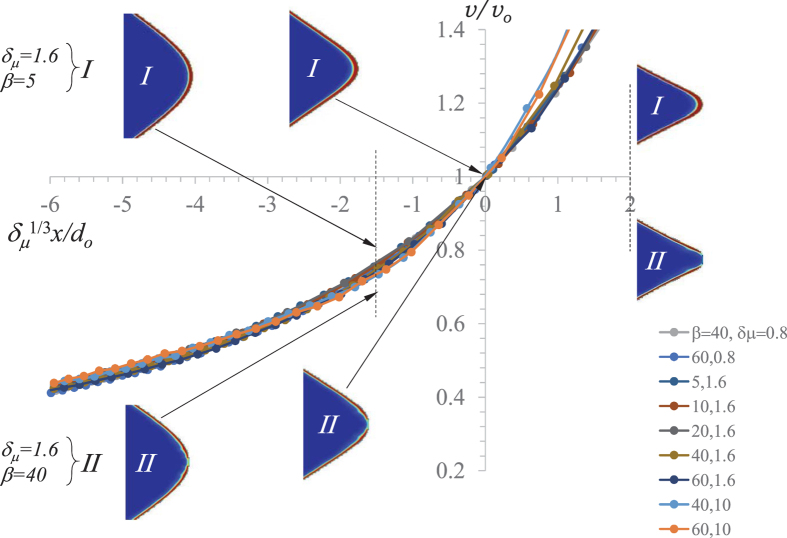
Velocity *v* of the tip as a function of its axial position *x* along the emission process for different values of *β* and *δ*_*μ*_. The origin of *x* axis is the position at which *v* = *v*_*o*_. The snapshots illustrate the form of the tip as well as the charge distribution at different stations in the self-similar collapse. The colors indicate the dimensionless volume charge density 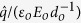 from 0 (blue) to 1 (red). The tip shape is similar for both values of *β*, while charge dwindling at the tip arises due to the limited charge relaxation in case II.

Figure 2 shows the self-similarity of this initial phase of the ejection process, as obtained from the numerical simulations. In this figure, we represent the tip velocity *v* as a function of its position *x*. This position and time t are made relative to the point where *v* = *v*_*o*_, and position is made dimensionless with 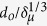 (this scaling has been obtained as the best fit from the analysis of the numerical data). As can be observed, the self-similarity of *v*(*x*) implies that increasing/decreasing *μ* makes both the characteristic axial length and time larger/smaller at the same pace.

### Bulk charge relaxation limit

At the initial stage of the drop deformation described above, the charge layer is relaxed over the drop free surface since any characteristic hydrodynamic time is much longer than the charge relaxation time *t*_*e*_ = *ε*_*i*_/*K* = *βt*_*o*_. However, the drop deformation eventually becomes an ultra-fast process which demands the transfer of ions from the bulk to the new free surface to sustain the electrostatic suction. At some instant during the collapse, the system passes by a critical station where electrokinetic effects become preponderant, the charge layer at the droplet tip “freezes”, and the surface charge density remains almost constant beyond that station. In other words, the axial velocity *v* at the tip becomes comparable to, and even greater than, that of the ions under the applied electric field. The limited mobility of ions prevents the further increase of the surface charge density in that region. In fact, if charge relaxation were sufficiently fast to keep the bulk quasi-neutral during the process, the droplet’s tip would collapse following a very different deformation path^4^.

**Figure 3:**
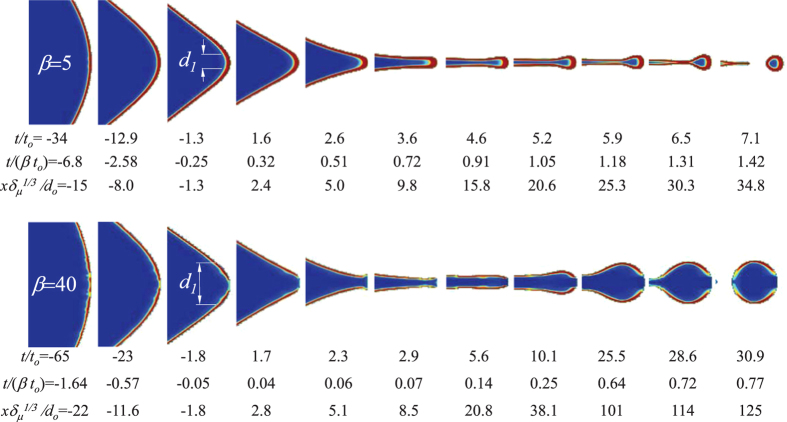
Images of the droplet ejection obtained from simulations with *β* = 5 and *β* = 40, and *δ*_*μ*_ = 1.6. The time was referenced to the instant when *v* = *v*_*o*_. The colors indicate the dimensionless volume charge density 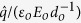 from 0 (blue) to 1 (red). Around *t*/(*βt*_*o*_) ~ −0.3, the surface charge density reaches its maximum value at a circumference with diameter *d*_1_. Beyond this stage, the tip moves faster than the bulk ions. The conical collapse up to *t*/*t*_*o*_ ~ 3.2 is geometrically self-similar.

Equation (1) establishes the order of magnitude of the tip’s axial velocity *v*. In addition, the characteristic hydrodynamic time *t*_*h*_ eventually becomes comparable to *t*_*e*_ as the ejection process accelerates, and therefore the local acceleration term becomes on the order of *ρv*_*o*_/*t*_*e*_. The size *d*_1_ and normal electric field *E*_*n*_ (surface charge density) of the “frozen cap” (Fig. 3) can be calculated from the balance between the local acceleration term, surface tension force, and electrostatic force at the station where *t*_*h*_ ~ *t*_*e*_, i.e.,


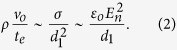


The above expression allows one to define the characteristic normal electric field *E*_*n*_ and length scale *d*_1_ as:


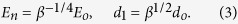


Figure 3 shows that the structure of the charge layer formed over the free surface remains almost constant once the radial scale of the tip goes below *d*_1_. An increase of *β* produces a decrease in the amount of ions able to reach the tip. This occurs because charge relaxation slows down as *β* increases, while the collapse of the drop tip proceeds at a speed *v*_*o*_ independent of that parameter. In particular, a disk completely depleted of charge appears at the tip for *β* = 40. This part of the free surface is kept devoid of charge even after breakup because the whole process takes place in times much shorter than the charge relaxation time *t*_*e*_.

The collapse ends when the tip size reaches the scale *d*_*o*_ (see Fig. 3 for 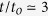). This condition verifies always beyond the stage where the charge layer freezes because 
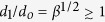
. The viscous term has been neglected in the balance (2). Therefore, our description of this process is valid as long as *d*_1_ is larger than, or at least comparable to, the capillary-viscous length 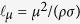, i.e., 
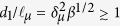
.

### Dynamics of the issued jet

The universal conical collapse described above gives rise to a nearly axial flow leading to the first-droplet emission. Now, we examine this axial flow to obtain the scaling laws for the droplet’s diameter and charge. Figure 3 shows a noticeable radial “bouncing” effect right after the collapse for *β* = 40. This occurs due to the restoring surface tension force appearing when the tip becomes depleted of charge, i.e., when the normal electric field on the tip fails to pull the liquid downstream with a sufficient force. Indeed, the larger *β*, the less favored the charge relaxation and the smaller the surface charge at the tip. Our simulations show that a front spot completely depleted of charge appears for 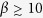.

The jet’s free surface is drawn by the applied electric field at a speed higher than that of the bulk, which hinders the capillary instability, and delays the breakup of the protojet produced during the collapse^19^ (Fig. 1). This leads to a rapid restructuring of the axial forces and the eventual arise of new scales as time goes on. Thus, the issued jet bulges significantly before breaking up for *β* = 40, instead of issuing an early fast drop as occurs for *β* = 5 (Fig. 3).

In all cases, the jet eventually reaches a stage where inertia, axial viscous stresses, electrostatic suction, and surface tension are balanced:


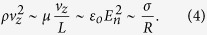


Here, *v*_*z*_, *L* and *R* are the characteristic axial velocity and length, and radius of the jet’s front portion, respectively. From the balances (4), and assuming that the surface charge density remains practically constant (*E*_*n*_ = *E*_*o*_*β*^−1/4^), one obtains the characteristic quantities:


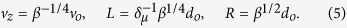


The scales of the emitted droplet’s diameter *d* and charge *q* immediately follow from (5):


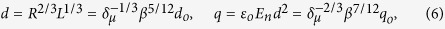


where 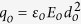. The scales of the Rayleigh limit charge, *q*_*R*_ = (*ε*_*o*_*σd*^3^)^1/2^, and the ratio *q*/*q*_*R*_ are


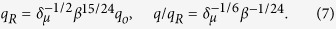


### Validation of the scaling laws

**Figure 4:**
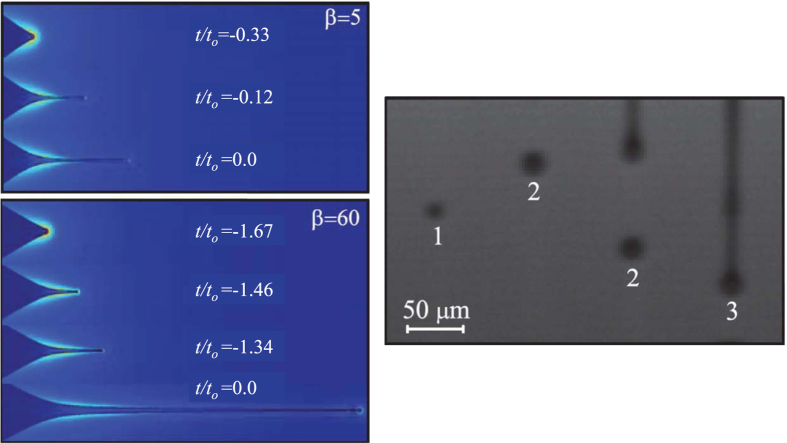
(Left) Numerical simulation of the droplet ejection for *β* = 5 and 60, and *δ*_*μ*_ = 5. The time was referenced to the instant when the jet breaks up. The color indicates the magnitude of the electric field (red/blue corresponds to high/low values of that quantity). Note that the time step in the first three frames is the same for the two cases. (Right) Experimental images of the first ejected droplets of 1-Octanol produced with 3000 kV. The four images correspond to the instants *t* = 0, 17.2, 21.5, and 30.1 *μ*s. The sequence shows the first, second, and the formation of the third droplet. The labels indicate the droplet number. The sharpness of the images is limited by both the low number of pixels (owing to the very high image acquisition speed) and the difficulties to get the optimum focus in such a local and fast event.

We conducted both numerical simulations and experiments to validate the above scaling analysis. Specifically, we examined the electrohydrodynamic response to a step change in the electric field magnitude of low-conductivity pendant drops surrounded by a gas. Details on the numerical and experimental methods can be found in *Methods*. Figure 4-left shows the numerical simulation of the droplet ejection for *β* = 5 and 60, and the same value of *δ*_*μ*_. The liquid volume set in motion increases significantly as *β* increases. For *β* = 60, the jet covers a distance about three times as that of the case *β* = 5 over a time interval around five times as that of *β* = 5. This result indicates that the tip velocity *v*_*z*_ decreases with *β* [Eq. (5)]. Figure 4-right shows the first, the second, and the formation of the third droplet in an experiment with 1-Octanol. The experiment shows that the first ejected droplet is significantly smaller than the rest of them. If the liquid volume ejected were replaced, then the transient regime analyzed here would eventually give rise to the steady mode of electrospray, which produces droplets of the same diameter.

**Figure 5:**
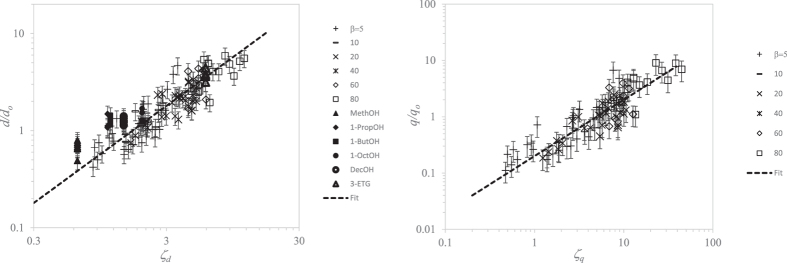
Measurements obtained from 125 numerical simulations (open symbols) and experiments conducted with the 6 liquids in Table 1 (solid symbols). The labels indicate the value of *β* in the numerical simulations, as well as the liquid used in the experiments. The error bars in the numerical data correspond to a relative uncertainty of 20%. We plot the diameters measured in all the experimental realizations to appreciate the scattering of the results. (Left) Dimensionless diameter *d*/*d*_*o*_ of the first ejected droplet versus 
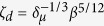
. The dashed line is the fit *d*/*d*_*o*_ = 0.6 *ζ*_*d*_ [Eq. (6)]. (Right) Electric charge *q*/*q*_*o*_ transported by the first ejected droplet as a function of 
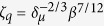
. The dashed line is the fit *q*/*q*_*o*_ = 0.2 *ζ*_*q*_ [Eq. (6)].

Figure 5-right shows the results obtained from 125 numerical simulations, varying *β* and *δ*_*μ*_ in the ranges [5, 80] and [0.045, 12.6], respectively. The figure also shows the diameters measured in the experiments. All these data are fitted by the linear expression *d*/*d*_*o*_ = 0.6 *ζ*_*d*_, where 
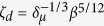
 [Eq. (6)]. Figure 5-right shows the numerical results for the electric charge transported by the first-ejected droplet (this quantity could not be measured experimentally) versus 
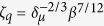
 [Eq. (6)]. The linear fit leads to *q*/*q*_*o*_ = 0.2 *ζ*_*q*_.

Collins *et al.*^7^ analyzed the formation of the first-issued droplet from an electrified parent drop by numerically solving the leaky-dielectric model, neglecting the time-dependent charge migration from the ligament bulk to its free surface. Therefore, their predictions are expected to deviate from our numerical and experimental results. Using their characteristic length 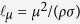, Eq. (6) writes


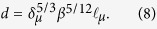


**Figure 6:**
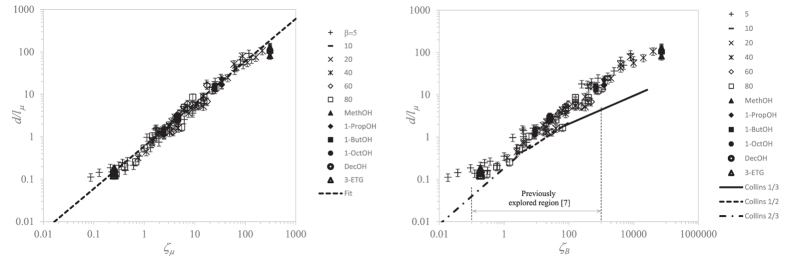
Dimensionless diameter 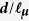 of the first ejected droplet versus 
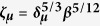
 (left) and 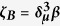 (right). The meaning of the symbols is the same as that in Fig. 5. The dashed line in the left-hand graph is the fit 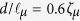 [Eq. (8)]. The solid, dotted, and dot-dashed lines in the right-hand graph are the scaling laws in [ref. 7, Fig. 3a].

Figure 6-left shows the numerical and the experimental data fitted by the linear relationship 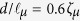, where 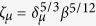 [Eq. (8)]. Our scaling law agrees remarkably well with both the numerical and experimental data over three and four decades of 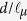 and *ζ*_*μ*_, respectively. In Fig. 6-right, we compare our experimental and numerical data with the scaling laws proposed in ref. 7. For this purpose, we make use of the same variable 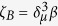 as that used in [ref. 7, Fig. 3a]. Their scaling laws deviate from our numerical simulations and experiments.

**Figure 7:**
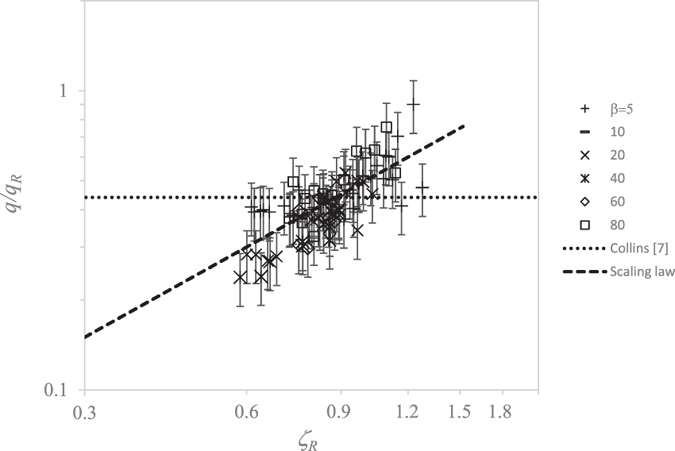
Ratio of electric charge *q* transported by the first ejected droplet to the Rayleigh limit *q*_*R*_ versus 
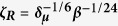
. The dashed line is the fit *q*/*q*_*R*_ = 0.6 *ζ*_*R*_ [Eq. (7)]. The dot-dashed line corresponds to the law *q*/*q*_*R*_ = 0.44 proposed by Collins *et al.*^7^. The meaning of the symbols is the same as that in Fig. 5.

Finally, Fig. 7 compares the present scaling law for the electric charge transported by the first-ejected droplet with that of ref. 7. The figure shows the droplet charge *q* relative to the Rayleigh limit *q*_*R*_ as a function of 
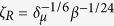
 [Eq. (7)]. The linear fit yields *q*/*q*_*R*_ = 0.5 *ζ*_*R*_, while the scaling law obtained in ref. 7 was *q*/*q*_*R*_ = 0.44. As can be observed, our scaling law satisfactorily agrees with the simulations.

To conclude, we have investigated the onset of electrospray through the formation of a conical cusp ejecting a very thin liquid ligament, akin to the disintegration of a liquid drop subject to an applied electric field. To understand this highly unsteady electrohydrodynamic process, we have focused our attention on the tiny drop formed in the ejected ligament front. Contrary to what happens in the *steady* cone-jet mode of electrospray, this process is determined by the onset of charge relaxation phenomena. The analyzed hydrodynamic process is so fast that charge migration towards the free surface is limited by the ion mobility, which becomes comparable to the free surface speed. As a consequence, the transport of electric charge cannot be simply modeled by a surface conservation equation with the Ohmic conduction term *KE*_*n*_, as can be made under steady conditions. Therefore, our study challenges prior models based on approximations, arguments or scaling laws for steady electrospray, which inherently implies nearly complete charge relaxation.

We propose a single (universal) set of scaling laws for the size and charge of the first-emitted droplet, which do not depend on the parameter region considered, and are valid within a range about three orders of magnitude larger than that explored in previous works^7^. These scaling laws have been validated from comparison with numerical simulations and experimental data. The atomization mode studied in this paper could be used to develop new ultra-sensitive analytic technologies because the described ejecta transport an exceptionally large electric charge per unit volume.

## Methods

### Numerical method

The Navier-Stokes equations were integrated in the liquid and gas domains with the Volume of Fluid (VoF) method. The surface tension stress is modeled as a volumetric force using the Continuum-Surface-Force approach. The electrohydrodynamic effects are accounted for from the electric and polarization volumetric forces arising in the liquid domain. The calculation of these forces relies on the knowledge of the volumetric charge distribution 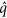 calculated from the equations


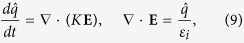


where *d*/*dt* denotes the time substantial derivative, *K* and *ε*_*i*_ are the constant bulk conductivity and permitivitty, and **E** is the electric field. The charge conservation equation can be obtained from its general form on the following three conditions: (i) thermal diffusion of ions is negligible, (ii) production-recombination of species is either negligible or at equilibrium, and (iii) the profiles of different ionic species are quasi-uniform except in thin boundary layers^20^. Conditions (i) and (iii) are closely related. In fact, if the distributions of ionic species are quasi-uniform in most of the liquid domain, then one can neglect both thermal diffusion and variations of conductivity in that region. Naturally, there must be a thin diffusive layer (the Debye layer) of thickness *λ*_*D*_ where the ionic concentration gradients must be considered. In that layer, thermal diffusion becomes relevant, and the conductivity is no longer constant. In our simulations, *λ*_*D*_/*d*_1_ ~ 10^−2^, and thus the resolution of that layer is not expected to affect significantly the results, but it would greatly increase the computing time. Both *λ*_*D*_ and *d*_1_ decrease as the electric conductivity increases. One expects *λ*_*D*_ to be much smaller than *d*_1_ in most practical situations. On the other side, the production-recombination of species can be neglected in unipolar injection or in solutions with strong electrolytes, which dissociate completely and quasi-instantaneously.

In order to estimate the uncertainty associated with the numerical method, simulations for fixed values of the governing parameters were conducted with meshes generated from different refinement procedures. To limit excessive computational expenses, we performed the study using meshes for which the variations of the first-droplet diameter were smaller than 20% compared to the values obtained using very fine meshes under the same conditions. The error bars in Fig. 5 correspond to that uncertainty.

### Experimental setup

A copper disk of 2 mm in radius and 0.7 mm in thickness was attached to the upper circular electrode. The disk was separated from the lower circular electrode by a distance of 10 mm. A drop hung from the disk surrounded by air, and with the triple contact line anchored to the disk edge. A constant voltage on the order of some kilovolts was applied to the upper electrode at a certain instant, while the lower electrode was kept at ground potential. More details on the experimental setup can be found elsewhere^8^. In each experimental realization, a first droplet of a few microns in diameter was ejected within a time interval on the order of 10^−5^ s. To visualize this singular event, digital images of the pendant drop tip were acquired at 235 000 frames per second with an ultra-high-speed camera (Photron, FASTCAM SA5) connected to high-magnification microscope (an OPTEM HR 10x magnification zoom-objective coupled to an Optem Zoom 70XL set of lenses). The images were processed to determine the diameter of the first issued droplet. Experiments were performed in the present work with the liquids listed in Table 1. The values of 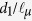 that establish the limit of our model are provided in this table as well.

## Additional Information

**How to cite this article**: Gañán-Calvo, A. M. *et al.* The onset of electrospray: the universal scaling laws of the first ejection. *Sci. Rep.* **6**, 32357; doi: 10.1038/srep32357 (2016).

## Figures and Tables

**Table 1:** Properties of the liquids used in the experiments.

Liquid	*ρ* (kg · m^−3^)	*σ* (N · m^−1^)	*μ* (Pa · s)	K (S · m^−1^)	*β*	*δ*_*μ*_	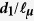
Methanol	795	0.021	0.00059	7.0 × 10^−6^	33.6	12.92	968.3
1-Propanol	803	0.0237	0.00194	9.0 × 10^−6^	20.33	3.92	69.48
1-Butanol	800	0.023	0.00254	6.0 × 10^−6^	17.84	3.36	47.83
1-Octanol	824	0.024	0.0089	6.6 × 10^−6^	10.34	0.95	2.916
n-Decanol	828	0.028	0.0118	1.08 × 10^−6^	7.93	1.48	6.175
3-Ethyl. glyc.	1120	0.0454	0.0402	4.0 × 10^−5^	23.7	0.199	0.1929

## References

[b1] Forbes T. P. Rapid detection and isotopic measurement of discrete inorganic samples using acoustically actuated droplet ejection and extractive electrospray ionization mass spectrometry. Rapid Commun. Mass Spectrom. 29, 19–28 (2015). 25462359 10.1002/rcm.7074

[b2] Melcher J. R. & Taylor G. I. Electrohydrodynamics: a review of the role of interfacial shear stresses. Annu. Rev. Fluid Mech. 1, 111–146 (1969).

[b3] Saville D. A. Electrohydrodynamics: The Taylor-Melcher leaky dielectric model. Annu. Rev. Fluid Mech. 29, 27–64 (1997).

[b4] Fontelos M. A., Kindelán U. & Vantzos O. Evolution of neutral and charged droplets in an electric field. Phys. Fluids 20, 092110 (2008).

[b5] Betelú S., Fontelos M. A., Kindelán U. & Vantzos O. Singularities on charged viscous droplets. Phys. Fluids 18, 051706 (2006).

[b6] Collins R. T., Jones J. J., Harris M. T. & Basaran O. A. Electrohydrodynamic tip streaming and emission of charged drops from liquid cones. Nature Phys. 4, 149–154 (2008).

[b7] Collins R. T., Sambath K., Harris M. T. & Basaran O. A. Universal scaling laws for the disintegration of electrified drops. PNAS 110, 4905–4910 (2013). 23487744 10.1073/pnas.1213708110 PMC3612677

[b8] Ferrera C., López-Herrera J. M., Herrada M. A., Montanero J. M. & Acero A. J. Dynamical behavior of electrified pendant drops. Phys. Fluids 25, 012104 (2013).

[b9] Oddershede L. & Nagel S. R. Singularity during the onset of an electrohydrodynamic spout. Phys. Rev. Lett. 85, 1234–1237 (2000). 10991520 10.1103/PhysRevLett.85.1234

[b10] Stachewicz U., Dijksman J. F., Yurteri C. U. & Marijnissen J. C. Experiments on single event electrospraying. Appl. Phys. Lett. 91, 254109 (2007).

[b11] Paine M. D. Transient electrospray behaviour following high voltage switching. Microfluid Nanofluid 6, 775–783 (2009).

[b12] Schnitzer O. & Yariv E. The Taylor-Melcher leaky dielectric model as a macroscale electrokinetic description. J. Fluid Mech. 773, 1–13 (2015).

[b13] Pillai R., Berry J. D., Harvie D. J. E. & Davidson M. R. Electrokinetics of isolated electrified drops. Soft Matter 12, 3310–3325 (2016). 26954299 10.1039/c6sm00047a

[b14] Gañán-Calvo A. M. Cone-jet analytical extension of Taylor’s electrostatic solution and the asymptotic universal scaling laws in electrospraying. Phys. Rev. Lett. 79, 217–220 (1997).

[b15] Higuera F. J. Flow rate and electric current emitted by a Taylor cone. J. Fluid Mech. 484, 303–327 (2003).

[b16] Gañán-Calvo A. M. The surface charge in electrospraying: Its nature and its universal scaling laws. J. Aerosol Sci. 30, 863–872 (1999).

[b17] Gañán-Calvo A. M., Rebollo-Muñoz N. & Montanero J. M. Physical symmetries and scaling laws for the minimum or natural rate of flow and droplet size ejected by taylor cone-jets. New J. Phys. 15, 033035 (2013).

[b18] Gañán-Calvo A. M. & Montanero J. M. Revision of capillary cone-jet physics: Electrospray and flow focusing. Phys. Rev. E 79, 066305 (2009). 10.1103/PhysRevE.79.066305 19658592

[b19] Gañán-Calvo A. M., Herrada M. A. & Montanero J. M. How does a shear boundary layer affect the stability of a capillary jet? Phys. Fluids 26, 061701 (2014).

[b20] Herrada M. A. *et al.* Numerical simulation of electrospray in the cone-jet mode. Phys. Rev. E 86, 026305 (2012). 10.1103/PhysRevE.86.026305 23005852

